# GPs’ perspectives on care models integrating medical and non-medical services in primary care—a representative survey in Germany

**DOI:** 10.1186/s12875-024-02693-x

**Published:** 2024-12-30

**Authors:** Wolfram J. Herrmann, Hendrik Napierala

**Affiliations:** Charité – Universitätsmedizin Berlin, Institute of General Practice and Family Medicine, Charitéplatz 1, Berlin, 10117 Germany

**Keywords:** Primary Care, Social Problems, Health Kiosk, Social Prescribing, Primary Care Centres

## Abstract

**Background:**

Health-related social problems are common in primary care. Different care models integrating medical and non-medical services in primary care have been tested and established nationally and internationally, such as social prescribing, social work in primary care, health kiosks and integrated primary care centres. The aim of our study was to explore the perspective of general practitioners (GPs) working in Germany on these four care models regarding their meaningfulness and if they would like to use them. Secondary objective was to explore factors influencing this assessment.

**Methods:**

We conducted a survey of a representative sample of GPs working in Germany. The questionnaire included questions on the assessment of the care models’ meaningfulness and whether the GPs would like to use them. The analysis was carried out descriptively and using linear regression.

**Results:**

One thousand four hundred thirty-nine GPs took part in the survey. Social prescribing and social work in primary care were rated as the most meaningful concepts. Over 65% of the GPs believed that using at least one of the care models would be beneficial. One in four GPs would even welcome the idea of integrating their practice into an integrated primary care center. Older age and male gender were associated with a more negative assessment of the care models.

**Conclusions:**

German GPs consider integrating medical and non-medical services in primary care to be meaningful, yet they are somewhat skeptical about its practical implementation in daily practice. However, younger GPs in Germany are significantly more receptive to these models.

**Trial registration:**

German Register of Clinical Studies (DRKS-ID: DRKS00032585; Registration Date: September 1, 2023).

## Background

In general practice consultations, health-related social problems such as loneliness, financial problems or problems in the family or at work come up regularly [1]. These problems can have a significant impact on the frequency and course of mental and somatic illnesses or can be caused by illnesses [2–4]. Health-related social problems disproportionately affect people of lower socioeconomic status, thereby increasing health inequalities [5]. Although health-related social problems are common in primary care, general practitioners (GPs) can only address them to a limited extent during consultations [6].

However, there is a large range of non-medical advice and services from governmental and non-governmental organizations available in many communities. Despite decades of local cross-sector collaboration efforts aimed at improving population health, a systematic review of 36 reviews reveals limited and mixed evidence of their effectiveness on health outcomes, health services, and resource use, with many influencing factors identified but sparse data linking them to successful outcomes [7]. Various care models integrating medical and non-medical services in primary care have been implemented or tested in pilot projects world wide. Four prominent models are social prescribing, in-practice social work services, health kiosks and integrated primary care centres. These approaches align with the concept of social interventions in primary care, which aim to address social issues but do not necessarily involve integrating various types of services. For example, Bloch and Rozmovits describe social interventions in primary care as including not only social prescribing but also screening for social needs and implementing equity-oriented practice changes [8].

Social prescribing is a concept developed in the United Kingdom which has been implemented broadly [9]. GPs have the opportunity to refer patients with psychosocial problems to so-called link workers, who talk to the patients and refer them to suitable local services in the community [10]. Predominantly non-controlled studies indicate positive effects of social prescribing while randomized controlled trials are mostly missing [11]. Nonetheless, social prescribing is gaining widespread international adoption [12].

In-practice social work services are more focused on classical social work with case management. In this model, a social worker offers social work services regularly in the GP practice [13]. These services are established in many countries [14].

Health kiosks are a care model that is located outside of the GP's office [15]. Originally health kiosks have been developed in Finland. Health kiosks are neighbourhood oriented and aim to be a low-threshold service. They offer group and individual services on health and social issues.

When it comes to integrated primary care centres, Powell Davies and others distinguish extended primary care from integrated primary care centres [16]. In extended primary care, GPs play a leading role and other medical-oriented services are also offered by nursing staff, for example. Integrated primary care centres go beyond this approach: They have a stronger community focus and integrate non-medical services such as social work; They are often not managed by a GP, but rather family doctor care is integrated on an equal footing with other professions. Integrated primary care centres usually include services such as social prescribing and in-practice social work services.

These four concepts do often overlap, e.g., integrated primary care centres may include social prescribing and social workers while social prescribing might refer to health kiosks. Thus, these are distinct concepts but not mutually exclusive. You can find a more detailed description of these models in Table 1.

**Table 1:** The four care models based on the explanations given in the questionnaire [17]

**Social prescribing**: With social prescribing, GPs have the opportunity to refer patients with health-related social problems to local services via a so-called link worker. If a patient with social problems, such as loneliness or problems at work, presents to a GP, a "social prescription" can be issued. The patient will then have one or more appointments with a link worker. Link workers are specially trained individuals with knowledge of the health and social care system. The link workers either come to the practice or work at other local facilities. The patient and Link Worker work together to create an action plan over the course of several meetings. The link worker supports the patient in accessing local services. This could be, for example, debt counseling, a gardening club, a gym or a choir. The aim of the concept is not only to solve individual problems, but also to strengthen the patient's problem-solving skills and strengthen neighborhood cohesion.
**In-practice social work services**: For In-practice social work services, a social worker usually comes to the practice at a fixed time (e.g. once a week in the afternoon) to conduct counseling sessions with patients. This mainly involves classic social work similar to the social services in a hospital. This can include, for example, submitting applications for long-term care insurance or advising on social benefits
**Health kiosks:** A health kiosk is a low-threshold service on health and social issues located in a neighborhood. The health kiosk can be visited without an appointment, but GPs can also refer patients to the health kiosk. Staff at the health kiosk provide advice on health issues. Courses, lectures and group offers are also available. The health kiosk cooperates with local doctors, hospitals and social institutions to which those seeking advice can be referred if necessary. The aim of the health kiosk is to improve the health status of patients and strengthen their health literacy.
**Integrated primary care centres:** In integrated primary care centers, all primary care is organized from a single source and tailored to regional needs. To this end, multi-professional teams are formed from healthcare, social care and other professions that work together as equals. Patients should receive continuous care under one roof and be supported in dealing with their problems. Group services, a local contact point such as a café and close cooperation with the neighborhood are typical elements of integrated primary care centers.

Although these four concepts have been implemented to varying degrees internationally, the perspectives of GPs on these models have received little research attention so far. Berett - Abebe report on a representative survey of GPs in the USA on collaboration with social work; However, they only asked about the presence of social workers in the practice and not about attitudes towards the care model [18]. In addition, the perspective of participating GPs was researched in pilot projects on such models [19, 20]. However, there is a lack of studies on the perspective of GPs regardless of their involvement in pilot projects.

In Germany, these models have not been widely implemented, with only a few pilot projects being carried out on a local scale. However, they are actively discussed in policy circles and the public discourse. This study centers on the perspectives of GPs working within the German healthcare system, which is characterized by a fragmented structure with distinct sectors for health and social care [21]. Germany’s healthcare is funded through a public insurance model (the Bismarckian model) and is divided into inpatient and outpatient care, each with independent organization and funding. Rehabilitation, nursing, and social care are funded and organized separately through various means and schemes. Primary care in Germany is GP-led, but GPs have no formal gatekeeping function. Community health nurses and other primary care providers are uncommon. Consequently, policy papers frequently highlight the need for more integrated services in Germany [22]. In Germany, there has so far been a representative survey of the population on health kiosks and primary care centres [23], but no representative survey of GPs on these care models. The aim of our study was therefore to representatively capture the perspective of the GPs working in Germany on these four care models by exploring how meaningful GPs assess these four concepts, if they would like to use them, and which characteristics of GPs and their practices influence their perspective.

## Methods

### Study design and sample

For our study, we conducted a representative survey among GPs in Germany. The participants (GPs) were selected using a random sample of 10,000 GPs. This covers almost a fifth of all GPs (*n* = 55,127) working in primary care in 2023 [24]. The random sampling was carried out by the National Association of Statutory Health Insurance Physicians from the Federal Medical Register after approval by the Federal Ministry of Health in accordance with Sect. 75 of Book X of the German Social Code (SGB X). We sent out a letter to these randomly chosen GPs on October 10, 2023, with a link including password and QR code to access a web-based survey. A reminder letter was sent on November 13, 2023.

### Questionnaire

The questionnaire asked about awareness of the care models [17] and then explained all models. The participants were asked to evaluate the concepts presented, whether they consider them useful for the German healthcare system and whether they would specifically like to implement them in their practice (‘I would like it if a link worker would come to my practice ( e.g. 1x/week) to refer my patients to suitable offers in the neighbourhood’, ‘I think it would be good to integrate my practice into a primary care centre’). In addition, the survey contained information about the person and the practice, including age, gender (man, woman, diverse, other), number of patients, social status of the practice environment and the proportion of patients with psychosocial problems. The questionnaire has been published as open access together with the study protocol [17].

### Analysis

Data analysis was performed using the statistical program R (version 4.0.2). The analysis was primarily descriptive. Age statements under 25 and over 85 were judged to be implausible. The representativeness of the participants was checked by comparing the region and gender with the original sample using the chi-square test.

In order to assess which individual and practice factors influence the assessment of the care models in terms of meaningfulness and usefulness, we calculated linear regression models assuming linearity of the 5-point Likert scale. The independent variable was the agreement as to whether this care model was meaningful and if the GPs would like it to be implemented in the GP practice. Dependent variables were age, gender (including age-gender interaction), proportion of psychosocial problems, number of patients treated per quarter, location of the practice and the social area of the practice.

Missing data has been reported in the descriptive statistics. In the regression models, participants with missing data for any of the covariates were excluded.

### Ethics and transparency

The study was conducted in accordance with the Declaration of Helsinki. Alle participants gave their written informed consent. The conduct of the survey was approved by the Ethics Committee of the Charité – Universitätsmedizin Berlin (EA2/154/23). In addition, the study was registered in the German Register of Clinical Studies (DRKS-ID: DRKS00032585). The study protocol and questionnaire were published prospectively [17].

## Results

### Description of the sample

For 6 GPs, there was no address available; for 161 GPs neither letter could be delivered. Of the remaining 9,833 GPs, 1,474 responded (15.0%), of which 1,439 gave informed consent to participate.

Six hundred three (45.8%) of the participants were female, 702 male, 13 diverse or with a different gender entry (cf. Table 2). The participants were in mean 53.0 (SD = 9.9) years old. 63.6% of the participants had more than 10 years of professional experience as a GP. 226 (17.2%) of the participants worked in a practice in an environment with a low social status, 223 (16.9%) in an environment with a high social status and almost two thirds in a mixed environment. The participants were evenly distributed across practices in rural communities, small towns, medium-sized towns and large cities. Most participants (54.5%) stated that they see more than 1000 patients per quarter. The proportion of patients with psychosocial problems was on average 40.6% (SD = 21.1%).

**Table 2:** Study participants

*N* = 1439	Participants	Missing
Gender		121
Male	603 (45.8%)	
Female	702 (53.3%)	
Diverse/Other	13 (1.0%)	
Age		140
Range	30–84	
Mean (Standard Deviation)	53.0 (SD = 9.9)	
Median	54	
Associations of statutory health insurance physicians (Region)		136
Baden-Württemberg	182 (14.0%)	
Bavaria	242 (18.6%)	
Berlin	41 (3.1%)	
Brandenburg	54 (4.1%)	
Bremen	5 (0.4%)	
Hamburg	19 (1.5%)	
Hesse	106 (8.1%)	
Mecklenburg-Western Pomerania	41 (3.1%)	
Lower Saxony	141 (10.8%)	
North Rhine	105 (8.1%)	
Rhineland-Palatinate	58 (4.5%)	
Saarland	21 (1.6%)	
Saxony	64 (4.9%)	
Saxony-Anhalt	35 (2.7%)	
Schleswig–Holstein	45 (3.5%)	
Thuringia	33 (2.5%)	
Westphalia-Lippe	111 (8.5%)	
Working experience as GP		122
Less than 5 years	220 (16.7%)	
5–10 years	259 (19.7%)	
10–20 years	392 (29.8%)	
More than 20 years	446 (33.9%)	
Social status of the practice neighbourhood		123
Low social status	226 (17.2%)	
Mixed social status	86 (65.9%)	
High social status	223 (16.9%)	
Size of the community		127
Rural (up to 5,000 inhabitants)	305 (23.2%)	
Small village (5,000–20,000 inhabitants)	354 (27.0%)	
Medium-sized town (20,000–100,000 inhabitants)	321 (24.5%)	
City (more than 100,000 inhabitants)	332 (25.3%)	
Number of GPs in the practice		125
Range	0.4–18	
Mean (Standard Deviation)	2.2 (SD = 1.5)	
Median	2	
Number of patients seen in a quarter (individually)		137
Up to 399	20 (2.2%)	
400—599	66 (5.1%)	
600—799	165 (12.7%)	
800—999	259 (19.9%)	
1,000 – 1,199	277 (21.3%)	
1,200 – 1,399	219 (16.8%)	
1,400 and more	287 (22.0%)	
Relative frequency of patients with psychosocial problems		124
Range	2%-100%	
Mean (Standard Deviation)	40.6% (SD = 21.1%)	
Median	34%	

The distribution across the statutory health insurance associations did not differ between the sample data set of the GPs and participants ( χ^2^ (240) = 255, *p* = 0.24), nor did the gender (χ^2^ (6) = 8, *p* = 0.24).

### Assessment of meaningfulness

**Fig. 1:**
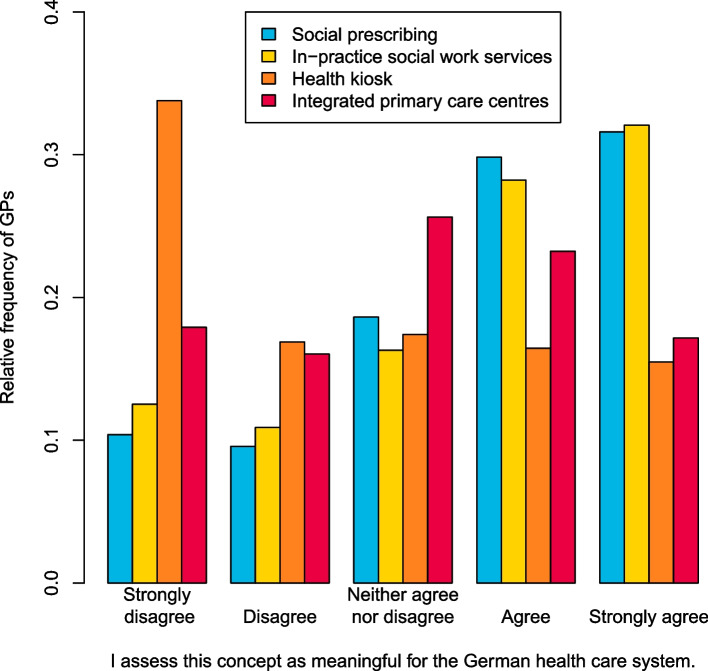
Assessment of the meaningfulness of models integrating medical and non-medical services in primary care for the German health care system by GPs

More than half of the participants considered social prescribing and in-practice social work to be meaningful, while almost half of the participants rated health kiosks as not meaningful. There was an ambiguous picture regarding the integrated primary care centres (cf. Fig. 1).

### Desired use

**Fig. 2:**
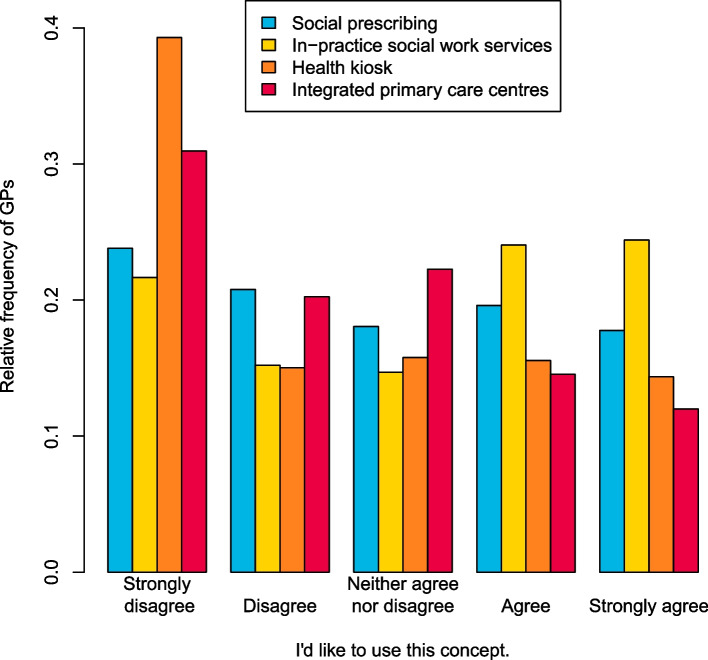
Assessment of usefulness models integrating medical and non-medical services in primary care by German GPs

The desire to use the respective care model in their own practice is met with less approval by the participants (cf. Fig. 2): Among the participants with information on all four models (*n* = 1,332), 133 (10.0%) could imagine to use all four models, and 873 (65.5%) agree with at least one care model. 254 (19.1%) do not agree with any of the four models. A third of the GPs would want to use social prescribing and a little less than half of the participants would like to offer in-practice social work in their practice. A quarter of participants would like to make referrals to a health kiosk. Almost a quarter of participants would like integrate their own practices into an integrated primary care centre.

### Predictors of the assessment of the care models

Younger GPs and women are more likely to rate the care models as meaningful, with the assessment between men and women becoming more similar as they get older (interaction). GPs that treat a higher proportion of patients with psychosocial problems more often consider in-practice social work services and integrated primary care centres to be useful. The environment of the practice and the number of patients have no influence. GPs with practices in areas with high social status more often consider social prescribing and in-office social work services to be meaningful (see Table 3).

**Table 3:** Predictors to assess the models as meaningful on a five-point-liker scale (from 1 to 5) with 95%-confidence intervals; a positive value means an increased assessment as meaningful

	Social prescribing	In-practice social work services	Health Kiosk	Integrated primary care centres
Intercept	4.34 (3.51;5.17)	4.49 (3.62;5.35)	3.68 (2.73;4.63)	4.13 (3.26;4.99)
Age in years/10	**-0.14 (-0.26; -0.03)**	**-0.25 (-0.37; -0.13)**	-0.13 (-0.26;0.00)	**-0.17 (-0.29; -0.05)**
Gender male	-0.51 (-1.3;0.27)	**-1.25 (-2.06; -0.43)**	**-1.35 (-2.24; -0.45)**	**-1.37 (-2.19; -0.56)**
Relative frequency of psychosocial problems /10	0.03 (0.00;0.07)	**0.08 (0.04;0.11)**	0.03 (-0.01;0.07)	**0.06 (0.02;0.09)**
Small village vs. rural	0.13 (-0.07;0.33)	0.06 (-0.14;0.27)	0.11 (-0.12;0.34)	0.14 (-0.07;0.35)
Medium-sized town vs. rural	0.1 (-0.1;0.31)	0.01 (-0.20;0.23)	0.14 (-0.09;0.37)	0.17 (-0.05;0.38)
City vs. rural	0.18 (-0.02;0.39)	0.09 (-0.13;0.3)	-0.02 (-0.26;0.21)	0.2 (-0.01;0.42)
400—599 patients/quarter vs. < 400	-0.18 (-0.74;0.37)	-0.06 (-0.64;0.52)	-0.21 (-0.85;0.43)	-0.37 (-0.96;0.21)
600—799 patients/quarter vs. < 400	0.01 (-0.5;0.51)	-0.03 (-0.56;0.49)	-0.31 (-0.89;0.27)	-0.27 (-0.8;0.26)
800—999 patients/quarter vs. < 400	-0.05 (-0.54;0.44)	-0.01(-0.52;0.5)	-0.29 (-0.86;0.27)	-0.27 (-0.78;0.24)
1,000 – 1,199 patients/quarter vs. < 400	-0.08 (-0.57;0.41)	-0.05 (-0.56;0.46)	-0.28 (-0.84;0.28)	-0.28 (-0.79;0.23)
1,200 – 1,399 patients/quarter vs. < 400	-0.14 (-0.64;0.36)	-0.10 (-0.62;0.42)	-0.34 (-0.91;0.23)	-0.28 (-0.79;0.24)
> = 1,400 patients/quarter vs. < 400	-0.4 (-0.89;0.09)	-0.33 (-0.84;0.18)	-0.56 (-1.12;0)	-0.38 (-0.9;0.13)
Neighbourhood with mixed social status vs low	0.02 (-0.17;0.22)	**0.25 (0.05;0.46)**	-0.17 (-0.39;0.05)	**-0.25 (-0.45; -0.04)**
Neighbourhood with high social status vs low	**0.31 (0.07;0.56)**	**0.46 (0.21;0.72)**	0.20 (-0.08;0.48)	0.05 (-0.2;0.31)
Interaction Age in years/10 * Gender male	0.06 (-0.08;0.21)	**0.20 (0.05;0.35)**	**0.20 (0.04;0.37)**	**0.22 (0.07;0.37)**

In terms of intention to use, younger GPs also have a more positive attitude towards the care models, with the exception of the health kiosk. Male general practitioners are less often interested in using the care models. The gap between men and healthcare providers of other genders again decreases with increasing age in terms of in-office social works services and integrated primary care centres (interaction). The location of the practice and the number of patients again have no influence. GPs in areas with mixed or higher social status are more likely to want to use social work services within the doctor's practice (see Table 4).

**Table 4:** Predictors regarding to want to use models on a five-point-liker scale (from 1 to 5) with 95%-confidence intervals; a positive value means an increased wish to use the model

	Social prescribing	In-practice social work services	Health Kiosk	Integrated primary care centres
Intercept	3.47 (2.56;4.38)	3.95 (3.01;4.90)	3.26 (2.30;4.22)	3.19 (2.31;4.08)
Age in years/10	**-0.20 (-0.32; -0.07)**	**-0.28 (-0.41; -0.15)**	-0.09 (-0.22;0.04)	**-0.17 (-0.3; -0.05)**
Gender male	**-1.09 (-1.94; -0.23)**	**-1.42 (-2.3; -0.53)**	**-1.04 (-1.95; -0.13)**	**-0.98 (-1.81; -0.15)**
Relative frequency of psychosocial problems /10	**0.10 (0.06;0.14)**	**0.12 (0.08;0.16)**	0.03 (-0.01;0.07)	**0.07 (0.03;0.11)**
Small village vs. rural	0.03 (-0.19;0.25)	0.06 (-0.17;0.29)	0.14 (-0.09;0.37)	0.15 (-0.06;0.36)
Medium-sized town vs. rural	0.13 (-0.1;0.35)	0.06 (-0.17;0.29)	0.18 (-0.06;0.42)	0.08 (-0.14;0.3)
City vs. rural	0.13 (-0.1;0.35)	0.05 (-0.18;0.28)	0.01 (-0.23;0.25)	0.13 (-0.09;0.34)
400—599 patients/quarter vs. < 400	-0.12 (-0.73;0.49)	0.02 (-0.61;0.66)	-0.09 (-0.74;0.56)	0.14 (-0.45;0.74)
600—799 patients/quarter vs. < 400	0.17 (-0.39;0.72)	0.05 (-0.52;0.62)	-0.16 (-0.74;0.42)	0.09 (-0.45;0.63)
800—999 patients/quarter vs. < 400	0.15 (-0.39;0.69)	0.2 (-0.36;0.76)	-0.15 (-0.72;0.42)	0.17 (-0.35;0.70)
1000—1.199 patients/quarter vs. < 400	0.24 (-0.29;0.78)	0.19 (-0.36;0.75)	-0.19 (-0.75;0.38)	0.19 (-0.33;0.71)
1.200—1.399 patients/quarter vs. < 400	-0.04 (-0.59;0.5)	0.00 (-0.56;0.57)	-0.29 (-0.87;0.28)	0.11 (-0.42;0.64)
>= 1.400 patients/quarter vs. < 400	-0.16 (-0.7;0.38)	-0.13 (-0.69;0.43)	-0.42 (-0.99;0.15)	0.14 (-0.39;0.66)
Neighbourhood with mixed social status vs low	0.04 (-0.17;0.25)	**0.25 (0.03;0.47)**	-0.22 (-0.44;0.01)	**-0.25 (-0.46; -0.05)**
Neighbourhood with high social status vs low	0.07 (-0.2;0.33)	**0.33 (0.05;0.60)**	0.05 (-0.23;0.34)	-0.09 (-0.35;0.17)
Interaction Age in years/10 * Gender male	0.15 (0.00;0.31)	**0.23 (0.07;0.40)**	0.15 (-0.02;0.32)	**0.17 (0.01;0.32)**

## Discussion

### Summary

German GPs rated social prescribing and in-practice social work services as the most meaningful models integrating medical and non-medical services in primary care, while health kiosks were overwhelmingly rejected. Over 65% of the GPs in Germany think it would be good to use at least one of the models, but there is no model that more than half of the participants would like to use. A quarter of the GPs would like to integrate their own practice into an integrated primary care centre.

### Strengths and Limitations

This survey is the first representative survey by GPs in Germany on models integrating medical and non-medical services in primary care. One strength is the representative random sample with an acceptable response rate. However, a major limitation is that a selection bias, i.e. a bias who responds to the survey, cannot be ruled out. The distribution across statutory health insurance associations and the gender of the participants, correspond to the distribution in the random sample. This suggests the absence of a significant selection bias. A second limitation is that GPs who were not aware of care models integrating medical and non-medical services in primary care had to rely on our descriptions of these models. Thus, our wording might have inadvertently influenced the GPs assessment.

### Comparison with existing literature

If you compare the results of our study with the representative survey of the population in Germany from the same year [23], it is noticeable that the proportion of the population who consider health kiosks and primary care centres to be meaningful is twice as high as the proportion among GPs in Germany: While 78% of the population thinks that primary care centres would improve health care, only 41% of GPs find them meaningful. 61% of the population think health kiosks would improve health care in Germany compared to 32% of the GPs who find them meaningful. It is noticeable that, as with the GPs surveyed, men and older people in the population are less likely to see the care models as useful, but this influence is significantly lower in the population than in our study. It is not clear why the public has more positive attitudes towards these models. One potential explanation might be, that the public stresses the individual potential positive impacts of these models, while GPs might focus more on potential work load for themselves in realising these models.

In this context, it is noteworthy that low-threshold models, which minimally interfere with GPs' work but remain under their responsibility—such as social prescribing and in-practice social work—are garnering greater interest for use. Conversely, the health kiosk model, which shifts responsibility away from GPs, is largely rejected by the majority from their perspective.

The strong influence of gender on the assessment of the care models is consistent with the representative survey of general practitioners' practices in the USA by Berrett -Abebe et al., in which male participants were less likely to have social workers employed in the practice [18]. Similar results were found by Döpfmer et al. with regard to the willingness to substitute GP tasks by non-medical staff, with greater acceptance among younger and female GPs [25]. Gisbert Miralles and others, however, found an increased delegation of activities by GPs among younger and male GPs [26].

Interestingly, personal factors such as age and gender are the dominant influences of the perceptions regarding these models. Structural factors such as the work load of the GP or the situation of the practice do not play a role. This contradicts the idea that a high work load might be a reason not to approve of such integrated models. We suggest to research the missing association between need and attitudes further with qualitative methods.

## Conclusions

Over 65% of the GPs working in Germany would like to use one of the four care models, but none of the models are of interest to the majority of the GPs. We think it is important to have a health care policy debate, if there is a need of different such models parallel to reach as many GPs and patients as possible. It is noticeable that a quarter of German GPs are interested in integrating their practice into an integrated primary care centre, with younger and non-male GPs being more willing to do so. This is particularly relevant because the definition of integrated primary care centres in our study had a multi-professional team at eye level as a core element which goes far beyond the current health care delivery model in Germany. It is therefore important to research which role future and young GPs would like to fill, whether they want to be in a team leading role as family doctors or in a role as part of a health and social care provision team that operates on an equal level.

## Data Availability

The study protocol and questionnaire were published prospectively at 10.5281/zenodo.8375384. The datasets used during the current study are available from the corresponding author on reasonable request until the dataset is published in an open data repository.
